# Supplementary material for: The Mycovirome in a Worldwide Collection of the Brown Rot Fungus Monilinia fructicola
Source: J Fungi (Basel). 2022 May 6;8(5):481. doi: 10.3390/jof8050481 (PMC9147972; doi:10.3390/jof8050481)
Supplement: Supplementary file 1 [file jof-08-00481-s001.zip › Supplementary Figure S1.pdf]

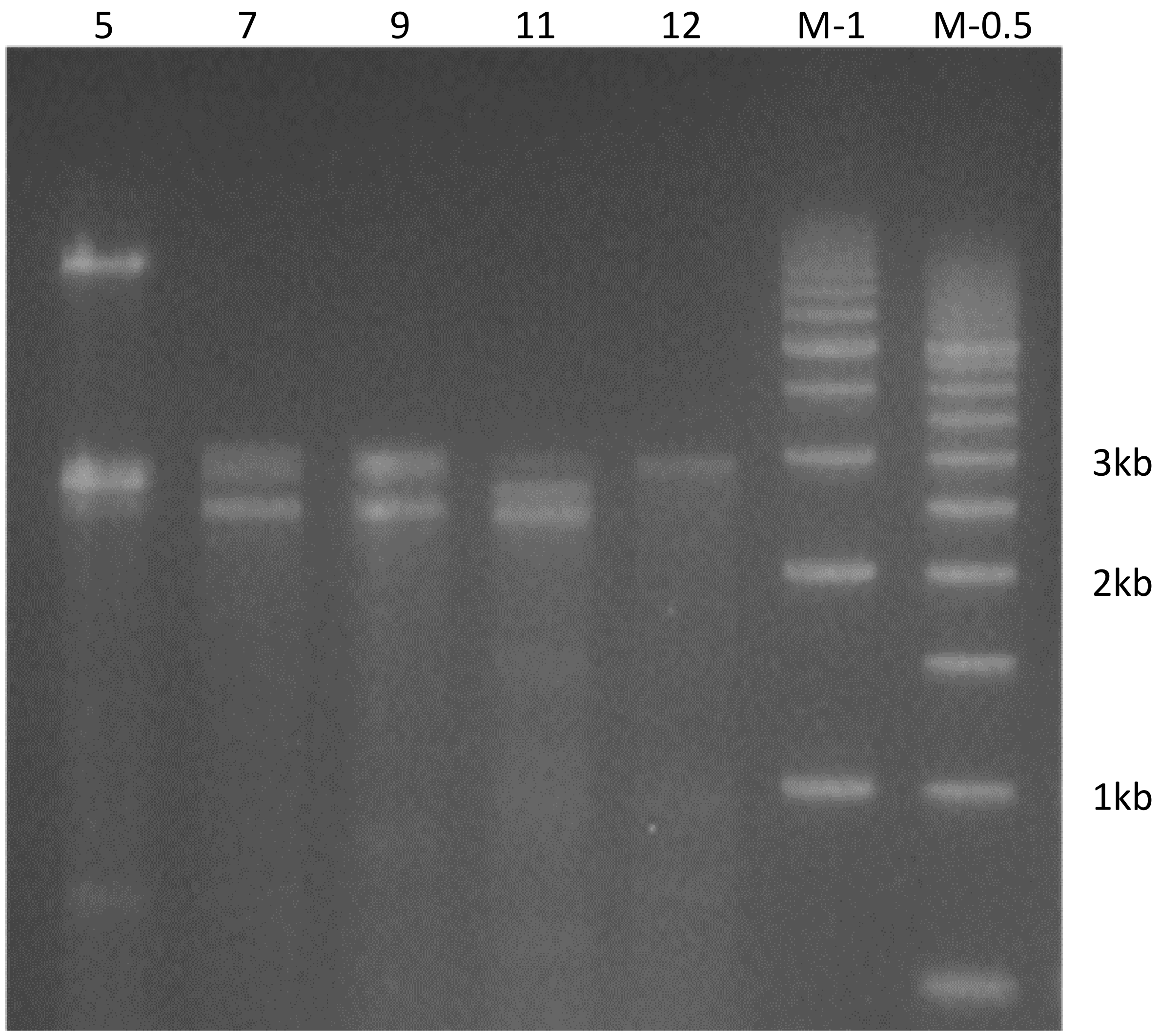

Supplementary Figure S1. Example of electrophoretic patterns of dsRNA extracted from pools of *Monilinia fructicola* isolates on a 1.5% agarose gel. The pool is indicated by the number on the top of each line. M = markers (M-1= 100-bp DNA Ladder, New England Bio-Labs; M-0.5= EZ Load™ 500 bp and 1 kb Molecular Ruler, Bio-Rad Laboratories).
